# Extended-Spectrum *β*-Lactamase (ESBL) Genotypes among Multidrug-Resistant Uropathogenic *Escherichia coli* Clinical Isolates from a Teaching Hospital of Nepal

**DOI:** 10.1155/2020/6525826

**Published:** 2020-04-15

**Authors:** Roshan Pandit, Balkrishna Awal, Sumesh Shreekhanda Shrestha, Govardhan Joshi, Basista Prasad Rijal, Narayan Prasad Parajuli

**Affiliations:** ^1^Department of Laboratory Medicine, Manmohan Memorial Institute of Health Sciences, Kathmandu, Nepal; ^2^National Public Health Laboratory, Teku, Kathmandu, Nepal; ^3^Kathmandu Center for Genomics and Research Laboratory (KCGRL), Kathmandu, Nepal; ^4^Department of Clinical Laboratory Services, Manmohan Memorial Medical College and Teaching Hospital, Kathmandu, Nepal

## Abstract

Urinary tract infections (UTI) represent the most common bacterial infections among patients visiting outpatient clinics of healthcare centers in Nepal. However, treatment of such infections is compounded by emergence and spread of multidrug-resistant uropathogens associated with extended-spectrum *β*-lactamases (ESBLs). In this study, we aimed to investigate the burden of antimicrobial resistance and occurrence of ESBL genes among clinical isolates of uropathogenic *Escherichia coli* at a tertiary care teaching hospital of Nepal. During the study period, we processed a total of 1,626 urinary tract specimens, isolated significant bacterial pathogens, and investigated their antimicrobial susceptibilities. *Escherichia coli* (*n* = 154), the predominant pathogen associated with UTI, was further investigated for the existence of ESBL enzymes by using conventional phenotypic as well as molecular approaches. Among suspected cases of UTI, we found that 15.2% were having UTI and female patients of the reproductive age group were more affected (*p* < 0.05). *Escherichia coli* (154, 62.1%) was the key uropathogen, and majority (∼64.9%) of them were multidrug resistant (MDR). Among MDR *E. coli* isolates, 40.3% were producing extended-spectrum *β*-lactamases (ESBLs). *bla-TEM* (83.8%), *bla-CTX-M* (66.1%), and *bla-SHV* (4.8%) were common ESBL genotypes. Nitrofurantoin, gentamycin, and imipenem were the most effective antibiotics for ESBL-producing *Escherichia coli* isolates. It indicates that the high rates of multidrug-resistant *Escherichia coli* are frequent causes of UTI in our hospital. Nitrofurantoin and aminoglycosides are the most useful first-line drugs to be used in the cases of UTI. We recommend the regular investigation of drug resistance among all isolates and develop a useful antibiotic prescription policy in our country.

## 1. Introduction

Urinary tract infection (UTI) represent a wide variety of clinical entities involving microbial invasion of any tissue of the urinary system from the renal cortex to the urethral meatus [1]. Every year, millions of people from all age groups are affected by UTI with a high risk of morbidity, mortality, and significant healthcare costs [2]. Furthermore, it is one of the most common infections in the community among the patients admitted to hospitals and is one of the principal causes of Gram-negative bacteremia [3, 4]. Females are more affected than males, and about 20% of women experience at least an episode of UTI during their life time and recurrence is very common [5, 6]. Therefore, accurate diagnosis and appropriate use of antimicrobials for treatment and prevention of urinary tract infections (UTIs) are necessary to reduce the burden as well as long-term consequences [7].

Etiological agents involved in urinary tract infection are much diverse, and the most commonly encountered microorganisms are Gram-negative Enterobacteriaceae including *Escherichia coli* [8]. The infections associated with these organisms are empirically treated with conventional antibiotics based on frequency of pathogens, local trends of antibiotic susceptibilities, and the illness severity [9]. However, increasing rates of antibiotic resistance and high recurrence rates have greatly reduced the therapeutic options for UTI in recent years [10]. Of particular concern, members of the family Enterobacteriaceae causing UTIs, including *E. coli* and *K. pneumoniae*, harboring acquired plasmids encoding extended-spectrum *β*-lactamases (ESBLs) are rising globally [11, 12]. These plasmids rapidly spread resistance to third-generation cephalosporins as well as other antibiotics [13, 14]. First detected in 1983, more than 300 variants of ESBLs have been identified in various members of the family Enterobacteriaceae and other nonenteric organisms [13]. Among various genotypes, *CTX-M*, *SHV*, *and TEM* have been described predominantly among the clinical strains of Enterobacteriaceae conferring broader antimicrobial resistance including *β*-lactams, fluoroquinolones, and aminoglycosides [15].

Early identification of ESBL-producing bacterial isolates causing clinical illness is highly important for appropriate treatment as well as for effective infection control in hospitals [11]. Increased rate of multidrug-resistant uropathogenic *Escherichia coli* among urinary tract infections has been reported previously from Nepal, and much of these studies were limited to phenotypic description of resistant bacteria [16, 17]. However, reports describing molecular types of ESBL-producing *Escherichia coli* causing urinary tract infections among the patients and their epidemiology are largely unknown. Therefore, it is very important for us to know the local epidemiology of urinary tract infections caused by ESBL-producing *Escherichia coli* and investigate the useful treatment alternatives in our settings. In this perspective, we aimed to determine the incidence, bacterial etiology of urinary tract infections, and genotypes of ESBL-producing multidrug-resistant *Escherichia coli* at a tertiary care health center of Kathmandu, Nepal.

## 2. Patients and Methods

This study was carried out at the Department of Clinical Laboratory Services of Manmohan Memorial Teaching Hospital, Kathmandu, Nepal, for six months (from February 2017 to July 2017). During the study period, a total of 1,626 urinary tract specimens were collected from patients presented with clinical suspicion of urinary tract infection and they were investigated for potential bacterial uropathogens.

## 3. Inclusion and Exclusion Criteria

Specimens representing the urinary tract infections among outpatients and inpatients attending Manmohan Memorial Teaching Hospital (MMTH) were included in the study. The clinical diagnosis of UTI was made by the respective unit physician in the presence of fever and/or any of the symptoms such painful micturition, increased frequency, burning micturition, and suprapubic pain/flank pain. All the urinary specimens (midstream urine and catheter-aspirated urine), aseptically collected before initiation of antimicrobial therapy, were included in the study. However, repeated samples from the same patient and those not fulfilling the criteria of the American Society for Microbiology were excluded.

## 4. Laboratory Procedure and Identification of Bacterial Uropathogens

Midstream urine and catheter-aspirated urine specimens were processed by standard microbiological methods without delay in the bacteriology laboratory of MMTH. They were processed semiquantitatively by inoculating 0.001 mL of the specimen (using a calibrated wire loop) onto the cystine lactose electrolyte deficient (CLED) agar, and the inoculated plates were incubated for 24 hours at 37°C in aerobic atmosphere. Growth of single organism with a count of ≥10^5^ colony-forming units (CFU)/mL was considered to represent the infection, and the organisms were identified using appropriate routine identification methods including colony morphology, Gram stain, and an in-house set of biochemical tests [18]. Among all isolates, the most predominant uropathogen, *Escherichia coli,* was further selected for the determination of antimicrobial susceptibility as well as detection of the multidrug-resistant (MDR) and extended-spectrum beta-lactamase- (ESBL-) producing strains.

## 5. Antimicrobial Susceptibility Testing

The antimicrobial susceptibility of *Escherichia coli* was determined by the disk diffusion method of modified Kirby–Bauer on the Mueller–Hinton agar (HiMedia Laboratories, India) following standard procedures recommended by the Clinical and Laboratory Standard Institute (CLSI), Wayne, USA [19]. Antibiotics included in the testing panel were amoxicillin (AMX 10 *µ*g), gentamycin (GEN 10 *µ*g), cotrimoxazole (COT 30 *µ*g), cefixime (CFM 5 *µ*g), Nitrofurantoin (NIT 300 *µ*g), ciprofloxacin (CIP 5 *µ*g), cefotaxime/ceftriaxone (CTX/CTR 30 *µ*g), ceftazidime (CAZ 30 *µ*g), piperacillin (PI 100 *µ*g), Piperacillin-tazobactam (PIT 100/10 *µ*g), tetracycline (TE 30 *µ*g), imipenem (IMP 30 *µ*g), and meropenem (MEM 30 *µ*g) (HiMedia Laboratories, India). Interpretations of antibiotic susceptibility results were made according to the zone size interpretative standards of the CLSI [19]. *Escherichia coli* ATCC 25922 was used as a control strain for antibiotic susceptibility.

## 6. Multidrug-Resistant (MDR) *Escherichia coli* and Potential ESBL Producers

In this study, *Escherichia coli* isolates resistant to at least one agent of three different classes of commonly used antimicrobial agents were regarded as multidrug resistant (MDR) [20]. If the zone of inhibition (ZOI) was ≤25 mm for ceftriaxone, ≤22 mm for ceftazidime, and/or ≤27 mm for cefotaxime, the isolate was considered a potential ESBL producer as recommended by the CLSI and further tested by confirmatory methods [19].

## 7. Combined Disk Test for Phenotypic Detection of ESBL

Presumptive ESBL-producing isolates by initial screening were emulsified with 4–6 ml of peptone water to adjust the inoculum density equal to that of 0.5 McFarland turbidity standards. Combination disk test (CDT), as recommended by the CLSI, was performed on all *Escherichia coli* isolates presumed to be ESBL producers. In this test, the cefotaxime (30 *µ*g) disk alone and in combination with clavulanic acid (cefotaxime + clavulanic acid, 30/10 *µ*g) disk were applied onto a plate of Mueller–Hinton agar (MHA) which was inoculated with the test strain and then incubated in ambient air for 16–18 hours at 35 ± 2°C. The isolate showing increase of ≥5 mm in the zone of inhibition of the combination discs in comparison to that of the cefotaxime disk alone was considered an ESBL producer [19].

## 8. Molecular Typing of ESBL Genes

All the phenotypic ESBL *Escherichia coli* isolates were subjected to molecular analysis for the confirmation of ESBL production. Molecular detection of *Escherichia coli* harboring ESBL genes (*bla-CTX-M*, *bla-TEM*, and *bla-SHV*) was carried out by conventional polymerase chain reaction (PCR) at the Kathmandu Center for Genomics and Research Laboratory (KCGRL), Kathmandu, Nepal. The methods for molecular detection of ESBL-producing isolates of *Escherichia coli* were similar as in our previous work [21].

## 9. Plasmid DNA Extraction and Amplification

For plasmid DNA extraction, a single colony of each potential ESBL-producing *Escherichia coli* was inoculated into Luria-Bertani broth and incubated till the logarithmic state. Extraction and purification of plasmid DNA of bacteria was carried out using a commercial kit (SureSpin Plasmid Mini kit from Genetix Biotech, Asia) following manufacturer's instructions. Purified DNA from bacterial isolates was used as a template to detect ESBL genotypes: *CTX-M*, *TEM*, and *SHV β*-lactamase genes. Primers for the amplification of ESBL genotypes (*bla-CTX-M*, *bla-TEM*, *and bla-SHV*) were designed and purchased from GeNei™, India, and their sequences are as listed in Table 1.

Polymerase chain reaction- (PCR-) based amplification of ESBL genes was carried out as the method previously described [22]. For instance, multiplex PCR was carried out to detect the plasmid genes for *SHV* and *CTX-M*, while conventional linear PCR was used for *TEM*-type ESBL genes. Amplification reactions were carried out in a DNA thermal cycler (CG) with the following thermal and cycling conditions: initial denaturation at 94°C for 3 minutes, denaturation at 94°C for 45 seconds of 35 cycles, annealing at 60°C for 30 sec of 35 cycles (for *bla-SHV* and *bla-CTX-M*) and 55°C for 30 sec of 35 cycles (for *bla-TEM*), extension at 72°C for 3 minutes of 35 cycles, and final extension at 72°C for 2 minutes. Previously confirmed *Klebsiella pneumoniae* clinical isolates possessing *bla-TEM*, *bla-SHV*, and *bla-CTX-M* genes were used as a positive control, and nuclease-free water was used as a negative control in each run of PCR.

After PCR amplification, 2.5 *μ*L of each reaction was separated by electrophoresis in 1% agarose gel for 30 min at 100 V (10 V/Cm) in 1 × TBE buffer. DNA was stained with ethidium bromide (1 *μ*g/ml), and the amplified DNA bands were visualized using a UV-transilluminator (Cleaver Scientific Ltd).

## 10. Data Analysis

The information regarding patient's profile and the results were entered into a computer program. Data analysis was carried out using the Statistical Package for Social Sciences (SPSS™) version 20.0 (IBM, Armonk, NY, USA) and presented in percentage base distribution. Data with *p* value of less than 0.05 (CI 95%) were regarded as significant.

## 11. Results

### 11.1. Patients with Urinary Tract Infection

Out of the total 1,626 urinary tract specimens from patients suspected of having UTI, 248 (15.2%) showed significant growth of at least one uropathogen confirming the urinary tract infection. Females (166, 66.9%) were the significant subgroup of patients (*p* < 0.05) affected with UTI, and most of them belonged to the age group 21–30 years. Incidence of UTI varied with different age group, gender, and type of patients (inpatients or outpatients) (Table 2).

### 11.2. Bacterial Uropathogens

Two hundred and forty eight bacterial uropathogens were recovered from a total of 1,626 patients with suspected UTI. Gram-negative bacteria (82.5%) were more common, and *Escherichia coli* (154, 62.1%) remained the predominant pathogen associated with UTI in all age groups. Other pathogens isolated from our UTI cases were *Staphylococcus aureus* (24, 9.7%), *Klebsiella pneumoniae* (22, 8.9%), *Enterococcus faecalis* (18, 7.3%), *Pseudomonas aeruginosa* (10,4.0%), and *Candida albicans* (4, 3.2%).

### 11.3. Antibiogram of *Escherichia coli*

**Figure 1:**
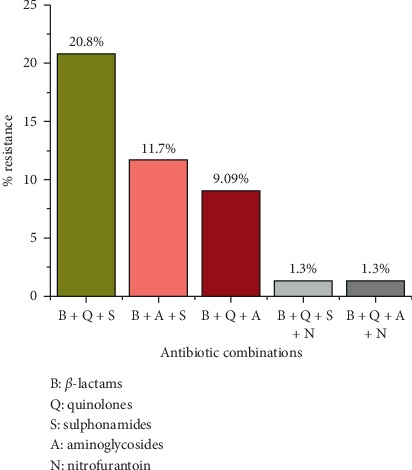
Pattern of antibiotic resistance for MDR *Escherichia coli* towards various antibiotic combinations. Antibiotic combinations and their relative percentage of resistance among uropathogenic *Escherichia coli* isolates.

Diverse pattern of antimicrobial susceptibilities was observed among the *E. coli* isolates. Nitrofurantoin (92.2%) and gentamycin (76.6%) were the most effective first-line therapeutic regimens for uropathogenic *E coli* isolates. Almost half of the isolates were resistant to cephalosporins and fluoroquinolones. Moreover, 64.9% (100/154) of *Escherichia coli* were found multidrug resistant. In combination, about 21% of the isolates were resistant to beta-lactam, quinolones, and sulfonamides, 12% were resistant to beta-lactam, aminoglycosides, and sulfonamides, and 9% were resistant to beta-lactam, quinolones, and aminoglycosides (Figure 1, Table 3).

### 11.4. ESBL *Escherichia coli*

About 40.3% (62/154) of our *Escherichia coli* isolates were confirmed as ESBL producers.

ESBL-producing *Escherichia coli* isolates were significantly more resistant to antibiotics as compared to nonproducers of ESBL (Table 3).

### 11.5. Genotype Distribution among ESBL *E coli*

**Figure 2:**
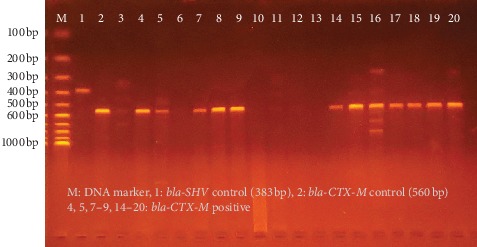
Agarose gel electrophoresis of amplified ESBL genes from MDR *Escherichia coli* isolates. Electrophoretic bands showing various genotypes of ESBL from MDR *Escherichia coli* isolates.

Sixty two isolates of *E. coli* were confirmed by molecular analysis of ESBL genes. Among the ESBL genotypes, *bla-TEM* (83.8%) was more common, followed by *bla-CTX-M* (66.1%) and *bla-SHV* (4.8%). More than half (54.8%) of the ESBL-producing *E. coli* isolates were coharboring *bla-TEM* and *bla-CTX-M* (Table 4, Figure 2).

## 12. Discussion

Urinary tract infection (UTI) continues to be the common clinical entity among the patients of the outpatient department and also represents one of the common nosocomial infections in Nepalese hospitals [6, 16]. However, the reported incidences and their epidemiology in Nepal are not consistent enough to reveal the actual scenario regarding the etiological spectrum and antimicrobial susceptibilities [5, 23–25]. In this laboratory-based study, we examined the organisms causing urinary tract infections and their antibiograms along with the production of extended-spectrum beta-lactamase enzymes by phenotypic and genotypic approaches. To the best of our knowledge, this report represents the first genotypic characterization of ESBL-producing uropathogens from the cases of urinary tract infections from Nepal.

Overall incidence of UTI in our study was quite low (15.2%) when compared to the previous reports from similar studies in Nepal [6, 16, 25]. The lower incidence in this study might be due to the prior use of antibiotics and infection due to slow-growing organisms or due to those organisms that were not able to grow on our routine culture media. In addition, more outpatients were found with UTI than inpatients. Concomitantly, significantly more females (66.9%) were found with UTI, as previously described elsewhere [5, 6, 25]. The higher occurrence of UTI in females of the reproductive age group in this study has been well supported by other studies [5, 26]. Furthermore, elderly males were found more affected by UTI in this study, as they might have bladder outflow obstruction and other chronic comorbid conditions.

We observed that Gram-negative bacteria were the most predominant (81.4%) organisms associated with our cases of UTI, and *Escherichia coli* (62.0%) was the major pathogen. Members of Enterobacteriaceae have been well described as the primary agents for UTI than other organisms in several studies. Higher incidence of *E. coli* seen in our study also resembled the results of previous studies from Nepal [16, 17, 25]. Although very low number of Gram-positive bacteria and yeasts were isolated in this study, they are also responsible for UTI in various studies [6, 27].

Antimicrobial resistance among uropathogenic bacterial species is one of the major findings of this study. *Escherichia coli*, the major uropathogen, was highly resistant to commonly used therapeutic drugs (beta-lactams, sulphonamides, quinolones, and aminoglycosides). Out of 154 *E. coli* isolates, 89.3% were resistant to ampicillin, 68.9% resistant to piperacillin, 50.4% to cefixime, 49.1% to cotrimoxazole, and 41.6% to ofloxacin. This finding is similar to the previous reports by Baral et al. [17], Neupane et al. [28], and Rijal et al. [26] from Nepal. Ampicillin and other oral cephalosporins were ineffective in our study, hence should be assessed before using as an empirical therapy. In addition to this, susceptibility findings of isolates against cephalosporins and quinolones show a substantial increase in their resistance, as reported by others [16, 29]. However, nitrofurantoin (92.2%) and gentamycin (76.6%) were effective against uropathogenic *E coli* strains. As stated by others too, these can be considered as the first-line therapeutic regimen for UTI cases in our settings [24, 26]. Carbapenems including imipenem and meropenem would be useful as secondary therapy for multidrug-resistant and complicated UTIs [30]. However, in the recent years, the emergence of urinary isolates with carbapenem resistance is further complicating the treatment of UTIs [31].

In this study, we found a high proportion of *E. coli* (64.9%) isolates to be multidrug resistant (MDR). Our findings on MDR bacteria in UTI cases are compatible with the reports from different parts of the world, including Nepal [17, 28, 32, 33]. These MDR *E. coli* strains are common in the study hospital as we previously reported the similar findings in pediatric UTI cases [34]. Furthermore, the most common MDR pattern among *E. coli* isolates was resistance towards beta-lactams + quinolones + sulphonamides (21%), which may be due to the production of hydrolytic enzymes (*β*-lactamases) by the bacteria [30]. Our finding suggests that the antibiotic treatment options for UTIs caused by *E. coli* have been severely challenged due to the resistance to commonly used antibiotics, leading to the situation relying only on certain reserve antibiotics.

Over the time, incidence and epidemiology of MDR and ESBL-producing uropathogenic *E. coli* have been continuously changing and higher rates are reported from developing countries [30]. In this study, 40.3% of the *E. coli* isolates were found as ESBL producers, and the patients over age of 50 years were found with higher incidence of ESBL *E coli.* The rate of ESBL in this study is very high when compared to the reported rates from previous studies [17, 29]. However, the rate of ESBL-producing uropathogenic *E coli* in Nepal has been constantly rising, and recently, Neupane et al (33.2%) and Bhandari et al (38.6%) have documented an increased rate of ESBL producers from UTI cases [28, 35]. From international perspectives, similar rates of ESBL-producing *E. coli* are also reported by Jena et al (41.07%) from India [32], Masud et al (40.9%) from Bangladesh [36], Moore et al (44%) from Cambodia [37], and Kizilca et al (41.4%) from Turkey [38]. However, the rates of ESBL-producing uropathogenic *E coli* from developed countries are very low as reported elsewhere [30, 39]. The reason behind the variation in ESBL-producing strains among studies might be attributable to the local antibiotic prescribing practices, extensive use of broad-spectrum antibiotics especially third-generation cephalosporins, and endemicity of drug-resistant pathogens in the locality.

Alongside, we observed diverse genotypes of ESBL among *E coli* isolates. In this study, *bla-TEM* (83.8%) was the most predominant genotype of ESBL among *E coli* isolates, which is well supported by a recent Indian study [32]. However, the *bla-CTX-M* gene has been described as the most common genotype of ESBL among enterobacteriaceae in several literatures [40–42]. In our previous report too, we found the dominance of the *bla-CTX-M* gene among ESBL-producing enterobacteriaceae from various clinical specimens [22]. Moreover, multiple occurrences of genes in a same organism were also noted, where *bla-TEM* + *bla-CTX-M* (54.8%) was common. These genes are usually present on the large plasmids accompanied with the genetic determinants conferring resistance towards various antimicrobials [43]. In this study too, ESBL-producing isolates were more resistant to cephalosporins and fluoroquinolones. However, nitrofurantoin and aminoglycosides proved to be the optimal first-line drugs in the cases of UTI caused by ESBL *E coli* in our study. This may be due to the restricted use of these drugs in our hospital setting, and nitrofurantoin is usually reserved to be prescribed only in cases of UTIs since it is excreted and concentrated in urine [29, 30]. Carbapenems can be reserved for severe cases of UTI where primary therapy is ineffective [30].

Infections caused by ESBL-producing organisms are a global problem. Mobile genetic elements contained in the bacterial species are easily transferable to other organisms in the vicinity [36]. Timely detection of the resistant strains along with their antimicrobial susceptibilities is very important for the effective management of UTI in the endemic regions. However, limited facilities of detection and poor understanding of such bugs in the developing counties are responsible for global dissemination of such pathogens [13, 15].

## 13. Limitations

Although it was a cross-sectional study, we were unable to evaluate the risk factors and outcomes of the patients with UTI. Molecular characterization of ESBL-producing *E. coli* strains with detailed analysis of phylogenetic outline would be more useful in understanding the epidemiology of ESBL *E. coli* in our setting. Furthermore, determination of minimum inhibitory concentration (MIC) of therapeutic antibiotics would be helpful for treatment and monitoring of the drug-resistant infections.

## 14. Conclusion

High burden of antimicrobial resistance and increased prevalence of ESBL-producing *Escherichia coli* associated with UTI are the major findings of this study. Diverse genotypes of ESBL *E. coli* along with resistance towards common antibiotics were observed. Nitrofurantoin and aminoglycosides were found as the most useful first-line drugs to be used in the cases of UTI in our setting. In this perspective, regular national-wide epidemiological surveillance of bacterial pathogens causing UTIs and their antimicrobial resistance would be useful in developing the treatment guidelines in our country.

## Figures and Tables

**Table 1:** Primers for the *bla-CTX-M*, *bla-TEM*, and *bla-SHV* genes.

Gene	Primers (5′-3′)	Amplicon size (bp)
*SHV*	F: 5`-GTCAGCGAAAAACACCTTGCC-3`	383 bp
	R: 5`-GTCTTATCGGCGATAAACCAG-3′	
*TEM*	F: 5`-GAGACAATAACCCTGGTAAAT-3`	459 bp
	R: 5′-AGAAGTAAGTTGGCAGCAGTG-3′	
*CTX-M*	F: 5′-GAAGGTCATCAAGAAGGTGCG-3′	560 bp
	R: 5′-GCATTGCCACGCTTTTCATAG-3′	

**Table 2:** Patients with urinary tract infection.

Age group (years)	Patients with urinary tract infection					
	Male (%)	Female (%)	*p*	Outpatients (%)	Inpatients (%)	*p*
0–10	4 (9.0)	8 (14.2)	0.318	10 (10.8)	2 (25.0)	0.245
11–20	2 (4.3)	22 (16.6)	**0.025**	24 (16.2)	0 (0.0)	**0.008**
21–30	4 (4.0)	46 (16.9)	**≤0.001**	48 (14.2)	2 (5.5)	0.108
31–40	6 (6.1)	36 (17.8)	**0.004**	40 (15.0)	2 (5.8)	0.112
41–50	8 (9.3)	10 (10.41)	0.500	16 (10.5)	2 (6.6)	0.400
51–60	18 (15.8)	18 (27.3)	**0.049**	32 (22.3)	4 (2.9)	0.073
61–70	8 (16.0)	10 (17.2)	0.536	12 (15.3)	6 (20.0)	0.377
71–80	22 (26.8)	8 (12.9)	**0.032**	20 (19.2)	10 (25.0)	0.292
>80	10 (26.3)	8 (33.3)	0.377	16 (4.2)	2 (8.3)	**0.004**

**Table 3:** Antibiotic susceptibilities of ESBL-producing and nonproducing uropathogenic *E. coli* isolates.

Antimicrobials	*Escherichia coli* urinary isolates			*p*
	Total	ESBL producers (*n* = 62)	ESBL nonproducers (*n* = 92)	
	Susceptible (%)	Susceptible (%)	Susceptible (%)	
Ampicillin	18 (11.7)	0 (0.0)	16 (17.3)	**0.005**
Piperacillin	48 (31.1)	8 (12.9)	40 (43.4)	**≤0.001**
Cefixime	78 (50.6)	0 (0.0)	78 (84.8)	**≤0.001**
Cefotaxime	82 (53.2)	4 (6.4)	78 (84.8)	**≤0.001**
Ceftazidime	84 (54.5)	4 (6.4))	80 (86.9)	**≤0.001**
Gentamycin	118 (76.6)	50 (80.6)	68 (73.9)	0.220
Cotrimoxazole	80 (51.9)	32 (51.6)	48 (52.1)	0.538
Nitrofurantoin	142 (92.2)	56 (90.3)	86 (93.4)	0.336
Ofloxacin	90 (58.4)	22 (35.4)	68 (73.1)	**≤0.001**
Imipenem	146 (94.8)	56 (90.3)	90 (97.8)	**0.047**
Meropenem	136 (88.3)	52 (83.8)	84 (91.3)	**0.018**

**Table 4:** Distribution of ESBL genotypes among uropathogenic *Escherichia coli* (*n* = 62).

ESBL genotypes	Frequency	%
*bla-CTX-M*	41	66.1
*bla-TEM*	52	83.8
*bla-SHV*	3	4.8
*bla-CTX-M* + *bla-TEM*	34	54.8

## Data Availability

All data generated during the study are included in this manuscript.

## Abbreviation

ATCC: American Type Culture Collection
ASM: American Society for Microbiology
*E coli*: *Escherichia coli*
CLSI: Clinical and Laboratory Standard Institute
ESBL: Extended-spectrum beta-lactamases
MDR: Multidrug resistant
MHA: Mueller–Hinton agar
*CTX-M*: Cefotaximase-Munich
*TEM*: Temoniera
*SHV*: Sulfhydryl variant
UTI: Urinary tract infection.
